# Biomechanical analysis of cervical myelopathy due to ossification of the posterior longitudinal ligament: Effects of posterior decompression and kyphosis following decompression

**DOI:** 10.3892/etm.2014.1557

**Published:** 2014-02-18

**Authors:** NORIHIRO NISHIDA, TSUKASA KANCHIKU, YOSHIHIKO KATO, YASUAKI IMAJO, YUICHIRO YOSHIDA, SYUNICHI KAWANO, TOSHIHIKO TAGUCHI

**Affiliations:** Department of Orthopedic Surgery, Yamaguchi University Graduate School of Medicine, Ube, Yamaguchi 755-8505, Japan

**Keywords:** ossification of the posterior longitudinal ligament, cervical myelopathy, finite element method

## Abstract

Cervical ossification of the posterior longitudinal ligament (OPLL) results in myelopathy. Conservative treatment is usually ineffective, thus, surgical treatment is required. One of the reasons for the poor surgical outcome following laminoplasty for cervical OPLL is kyphosis. In the present study, a 3-dimensional finite element method (3D-FEM) was used to analyze the stress distribution in preoperative, posterior decompression and kyphosis models of OPLL. The 3D-FEM spinal cord model established in this study consisted of gray and white matter, as well as pia mater. For the preoperative model, 30% anterior static compression was applied to OPLL. For the posterior decompression model, the lamina was shifted backwards and for the kyphosis model, the spinal cord was studied at 10, 20, 30, 40 and 50° kyphosis. In the preoperative model, high stress distributions were observed in the spinal cord. In the posterior decompression model, stresses were lower than those observed in the preoperative model. In the kyphosis model, an increase in the angle of kyphosis resulted in augmented stress on the spinal cord. Therefore, the results of the present study indicated that posterior decompression was effective, but stress distribution increased with the progression of kyphosis. In cases where kyphosis progresses following surgery, detailed follow-ups are required in case the symptoms worsen.

## Introduction

Ossification of the posterior longitudinal ligament (OPLL) is recognized as a common clinical entity that results in compression myelopathy of the cervical spinal cord. Since conservative treatment for severe myelopathy caused by OPLL is usually ineffective, surgical treatment is selected for the majority of cases. Decompressive surgical procedures for OPLL-associated cervical myelopathy are divided into those using an anterior or a posterior approach. Iwasaki *et al* (1) identified that laminoplasty was effective and safe for the majority of OPLL patients that had an occupying ratio of OPLL <60% and with plateau-shaped ossification. However, neurological outcomes following laminoplasty for cervical OPLL were poor to fair in patients with an occupying ratio of >60% and/or hill-shaped ossification (1). One of the factors associated with poor surgical outcomes following laminoplasty for cervical OPLL is kyphosis (1,2).

Clinical results from patients treated with the posterior approach have been previously reported (1,2). However, to date, there have been no studies focusing on the stress distributions of posterior decompression for cervical OPLL and the effects of kyphosis. In the present study, a 3-dimensional finite element method (3D-FEM) was used to analyze the stress distributions of posterior decompression, as well as kyphosis, in a spinal cord with cervical OPLL and hill-shaped ossification.

## Materials and methods

### Spinal cord models

**Figure 1:**
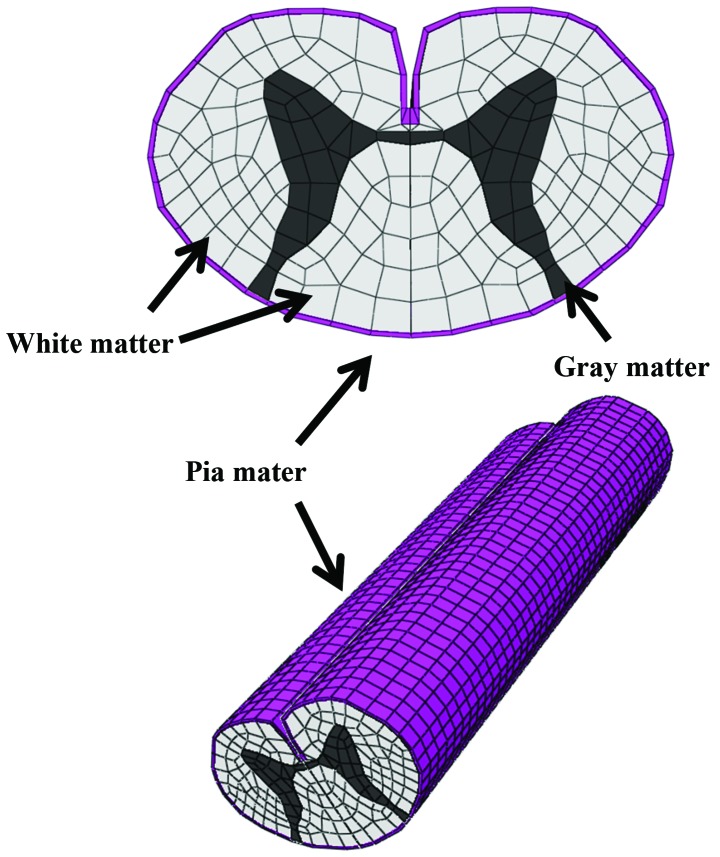
3D-FEM of the spinal cord consisting of gray matter, white matter and pia mater. 3D-FEM, 3-dimensional finite element model.

Abaqus 6.11 (Dassault Systèmes Simulia Corporation, Providence, RI, USA) finite element package was used for FEM simulation. The 3D-FEM spinal cord model established in this study consisted of gray and white matter, as well as pia mater (Fig. 1). To simplify calculations in the model, the denticulate ligament, dura and nerve root sheaths were not included. The pia mater was included since it has been previously identified that the spinal cord with and without this component shows significantly different mechanical behavior (3). The spinal cord was assumed to be symmetrical around the mid-sagittal plane; therefore, only half the spinal cord required reconstruction and the whole model was integrated by mirror image. For computed tomography-myelography (CTM) measurement, the vertical length of the spinal cord was two vertebral bodies (~40 mm).

**Figure 2:**
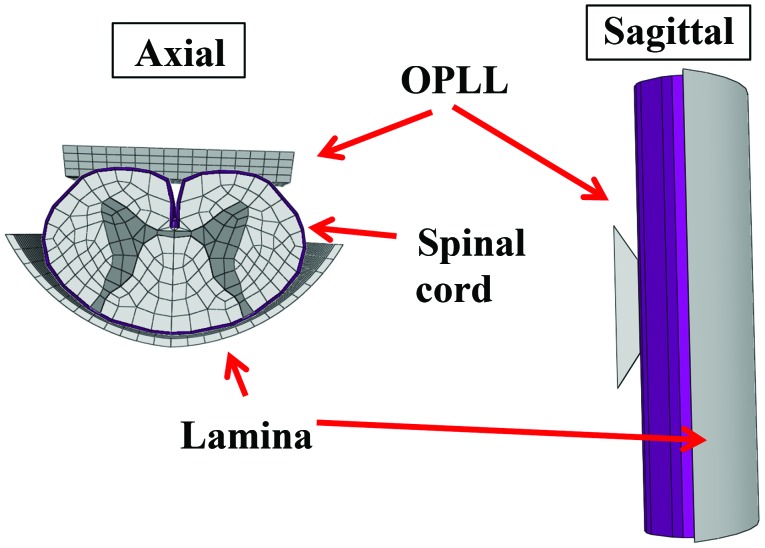
Lamina model with hill-shaped OPLL established at the rear of the spinal cord (axial and sagittal view). OPLL, ossification of the posterior longitudinal ligament.

The lamina model was established by measuring CTM and magnetic resonance imaging (MRI) and simulated cervical OPLL. A rigid, wide trapezium body with a slope of 30° was used to simulate cervical OPLL by measuring the MRI of paper (Fig. 2) (1).

### Mechanical properties

The spinal cord consists of three distinct materials referred to as white matter, gray matter and pia mater. The mechanical properties (Young’s modulus and Poisson’s ratio) of the gray and white matter were determined using data obtained by the tensile stress strain curve and stress relaxation under various strain rates (4,5). The mechanical properties of pia mater were obtained from previous literature (6). The mechanical properties of hill-shaped ossification and lamina were stiff enough for the spinal cord to be pressed. Based on the assumption that no slippage occurs at the interfaces of white matter, gray matter and pia mater, these interfaces were glued together. Since there are no data on the friction coefficient between the lamina and spinal cord, this was assumed to be frictionless. Similarly, the coefficient of friction between the hill-shaped ossification and spinal cord was assumed to be frictionless at the contact interfaces.

The spinal cord, hill-shaped ossification and lamina model were symmetrically meshed with 20-node elements. The total number of isoparametric 20-node elements was 11,542 and the total number of nodes was 66,513.

### Compression

In a biomechanical study of static compression of cervical myelopathy due to OPLL, Kato *et al* (7) reported that a critical point may exist between 20 and 40% compression of the anterior-posterior diameter of the spinal cord. For the preoperative model, compression was simulated by cervical OPLL with hill-shaped ossification. The lamina was fixed in all directions and 30% anterior static compression of the anterior-posterior diameter of the spinal cord (median, 20–40%) was applied by OPLL (1,7). For the posterior decompressive model, the lamina was shifted back to prevent contact with the spinal cord under the application of anterior static compression. For the kyphosis model, the spinal cord was studied at 10, 20, 30, 40 and 50° kyphosis. The extent of stretching the spinal cord was 20% of the length of the spinal cord indicated in a previous study (8).

In total, seven compression combinations were evaluated and in each cross-section the average von Mises stress was recorded the color-coded made for each stress in the spinal cord.

## Results

### Stress distribution in the three models

**Figure 3:**
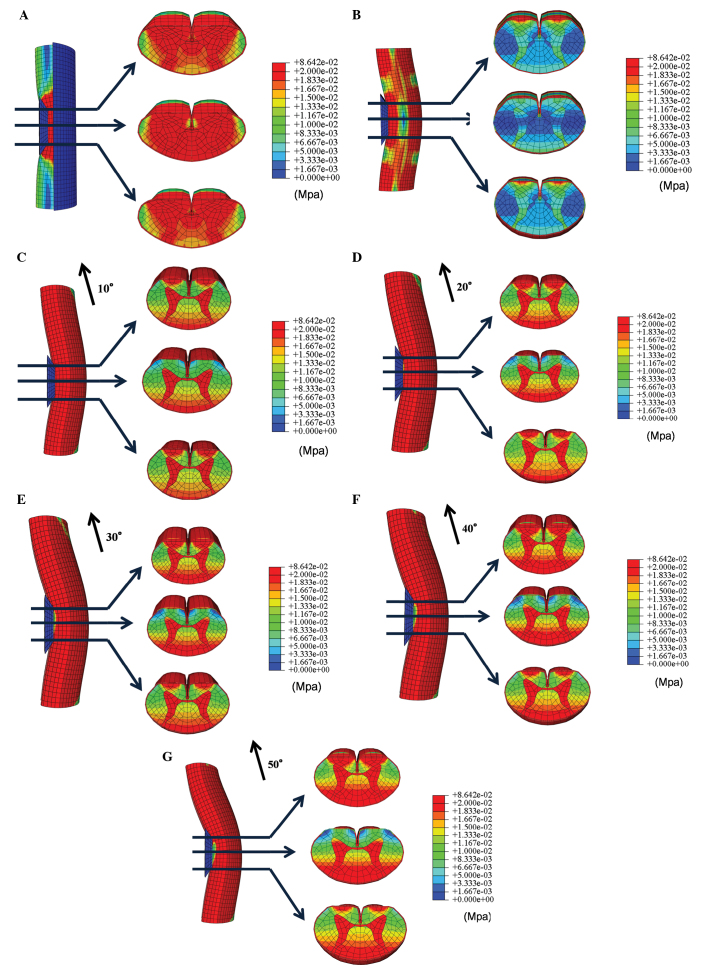
Stress distributions under proximal, central and distal anterior compression of the spinal cord by OPLL are shown in the (A) preoperative, (B) posterior decompression and (C–G) kyphosis models at 10, 20, 30, 40 and 50° kyphosis. OPLL, ossification of the posterior longitudinal ligament.

In the preoperative model, high stress distributions were observed in all axial levels of the spinal cord following anterior static compression (30% of the anterior-posterior diameter of the spinal cord) by cervical OPLL with hill-shaped ossification (Fig. 3A).

In the posterior decompression model, stresses from anterior compression of the spinal cord were lower compared with those observed in the preoperative model. However, stresses in the anterior funiculus slightly increased (Fig. 3B).

For the kyphosis model, stress distribution increased in the anterior funiculus, posterior funiculus and the gray matter in proximal and distal OPLL. The stress distribution also increased in the posterior funiculus and the gray matter in the center of OPLL. Furthermore, increasing the angle of kyphosis resulted in increased stress on the spinal cord (Fig. 3C–G).

## Discussion

The development of myelopathy significantly affects the prognosis of patients with OPLL in the cervical spine. Cervical OPLL is treated by anterior decompression and spinal fusion or laminoplasty. Tani *et al* identified that postoperative neurological deterioration occurred following posterior surgery. The authors indicated that one of factors of neurological deterioration affectedto decrease in the lordosis of the cervical spine (9).

Masaki *et al* reported that patients with a poor outcome following laminoplasty showed larger segmental mobility of the vertebrae prior to and following surgery. The authors hypothesized that laminoplasty in patients with massive OPLL may not lead to sufficient posterior shift of the spinal cord, resulting in persistent anterior impingement of the spinal cord by OPLL. In cases where substantial segmental mobility remains following surgery, it is possible that damage to the injured spinal cord continues to progress (10).

Iwasaki *et al* reported that a postoperative change in cervical alignment was observed in 18% of cases. Their study indicated that postoperative changes in cervical alignment may be a reflection of dynamic instability. A poor surgical outcome following laminoplasty was indicated by newly developed cervical kyphosis (1).

Using this prior knowledge, the present study investigated whether the development of kyphosis of the spinal cord following anterior compression was associated with changes in stress distribution. The aim was to develop a 3D-FEM spinal cord model that simulated the clinical situation and analyzed the clinical condition of the patient. Similarly to previous studies by Kato *et al* (7,11,12), Li *et al* (13,14) and Nishida *et al* (15,16), bovine spinal cord was used in the current analytical model since it was impossible to obtain fresh human spinal cord. The mechanical properties of the spinal cord used in the present study were similar to those used in earlier studies (4–6). Li *et al* identified that it was reasonable to use the mechanical properties of the bovine spinal cord since the brain and spinal cord of cattle and humans show similar injury changes (14). For the purpose of the present study, it was therefore assumed that the mechanical properties of the spinal cord from these two species were similar. Persson *et al* (3) reported on the division of the spinal cord into pia mater and white and gray matter. The authors demonstrated that the presence of pia mater had a significant effect on spinal cord deformation. Therefore, pia mater was included in the current model in order to accurately simulate the clinical situation.

In the present study, stress distribution in the spinal cord increased following static compression by cervical OPLL with hill-shaped ossification. Stress distribution in the spinal cord decreased in the posterior decompression model, demonstrating the effectiveness of this approach. However, in the kyphosis model, stress distribution increased with increased angles of kyphosis. Thus, when segmental mobility remains and cervical alignment changes following posterior decompression, damage to the spinal cord and the progression of symptoms are likely to occur.

In conclusion, stress analyses were conducted in models of preoperative compression, posterior decompression and kyphosis following posterior decompression by cervical OPLL with hill-shaped ossification.

Posterior decompression was shown to be effective, however, stress distribution increased with the progression of kyphosis, indicating that symptoms are likely to worsen. In cases where kyphosis has progressed following surgery, particularly those in which the angle of kyphosis is large, detailed follow-ups should be conducted in case the symptoms worsen.

## References

[1] Iwasaki M, Okuda S, Miyauchi A, Sakaura H, Mukai Y, Yonenobu K, Yoshikawa H (2007). Surgical strategy for cervical myelopathy due to ossification of the posterior longitudinal ligament: Part 1: Clinical results and limitations of laminoplasty. Spine (Phila Pa 1976).

[2] Chiba K, Ogawa Y, Ishii K, Takaishi H, Nakamura M, Maruiwa H, Matsumoto M, Toyama Y (2006). Long-term results of expansive open-door laminoplasty for cervical myelopathy - average 14-year follow-up study. Spine (Phila Pa 1976).

[3] Persson C, Summers J, Hall RM (2011). The importance of fluid-structure interaction in spinal trauma models. J Neurotrauma.

[4] Ichihara K, Taguchi T, Shimada Y, Sakuramoto I, Kawano S, Kawai S (2001). Gray matter of the bovine cervical spinal cord is mechanically more rigid and fragile than the white matter. J Neurotrauma.

[5] Ichihara K, Taguchi T, Sakuramoto I, Kawano S, Kawai S (2003). Mechanism of the spinal cord injury and the cervical spondylotic myelopathy: new approach based on the mechanical features of the spinal cord white and gray matter. J Neurosurg.

[6] Tunturi AR (1978). Elasticity of the spinal cord, pia, and denticulate ligament in the dog. J Neurosurg.

[7] Kato Y, Kanchiku T, Imajo Y (2010). Biomechanical study of the effect of the degree of static compression of the spinal cord in ossification of the posterior longitudinal ligament. J Neurosurg Spine.

[8] Henderson FC, Geddes JF, Vaccaro AR, Woodard E, Berry KJ, Benzel EC (2005). Stretch-associated injury in cervical spondylotic myelopathy: new concept and review. Neurosurgery.

[9] Tani T, Ushida T, Ishida K (2002). Relative safety of anterior microsurgical decompression versus laminoplasty for cervical myelopathy with a massive ossified posterior longitudinal ligament. Spine (Phila Pa 1976).

[10] Masaki Y, Yamazaki M, Okawa A (2007). An analysis of factors causing poor surgical outcome in patients with cervical myelopathy due to ossification of the posterior longitudinal ligament: anterior decompression with spinal fusion versus laminoplasty. J spinal Disord Tech.

[11] Kato Y, Kataoka H, Ichihara K (2008). Biomechanical study of cervical flexion myelopathy using a three-dimensional finite element method. J Neurosurg Spine.

[12] Kato Y, Kanchiku T, Imajo Y (2009). Flexion model simulating spinal cord injury without radiographic abnormality in patients with ossification of the longitudinal ligament: the influence of flexion speed on the cervical spine. J Spinal Cord Med.

[13] Li XF, Dai LY (2009). Three-dimensional finite element model of the cervical spinal cord. Spine (Phila Pa 1976).

[14] Li XF, Dai LY (2010). Acute central cord syndrome: injury mechanisms and stress features. Spine (Phila Pa 1976).

[15] Nishida N, Kato Y, Imajo Y, Kawano S, Taguchi T (2011). Biomechanical study of the spinal cord in thoracic ossification of the posterior longitudinal ligament. J Spinal Cord Med.

[16] Nishida N, Kato Y, Imajo Y, Kawano S, Taguchi T (2012). Biomechanical analysis of cervical spondylotic myelopathy: the influence of dynamic factors and morphometry of the spinal cord. J Spinal Cord Med.

